# Research on Multi-Physics Coupling Simulation for the Pulse Electrochemical Machining of Holes with Tube Electrodes

**DOI:** 10.3390/mi12080950

**Published:** 2021-08-11

**Authors:** Zhaolong Li, Bingren Cao, Ye Dai

**Affiliations:** 1 Key Laboratory of Advanced Manufacturing Intelligent Technology of Ministry of Education, Harbin University of Science and Technology, Harbin 150080, China; lizhaolong@hrbust.edu.cn; 2 School of Mechanical and Power Engineering, Harbin University of Science and Technology, Harbin 150080, China; 1920110144@stu.hrbust.edu.cn

**Keywords:** electrochemical machining, accuracy, stability, period, duty ratio, lateral gap, inlet pressure

## Abstract

Electrical parameters of the power supply are significant factors affecting the accuracy and stability of the electrochemical machining (ECM). However, the electric field, flow velocity and temperature in the machining area are difficult to measure directly under the influence of the power supply. Therefore, taking the film cooling hole as an example, the multi-physics coupling simulation analysis of the ECM is performed on the basis of Faraday’s law and fluid heat transfer mathematical model. The machining characteristics of the direct current and pulse ECM are compared through simulation. The results show that the pulse ECM improves the distribution of temperature and current density in the machining area. The period has little effect on the temperature, current density and side removal rate. The side removal rate increases with the increase of the duty ratio and lateral gap. Increasing of the duty ratio and decreasing of the lateral gap will increase the temperature and current density. Increasing the inlet pressure accelerates the frequency of renewal of heat and electrolysis products, which can reduce the single side gap. The experience of the ECM holes verifies the results of the simulation. The accuracy and stability of the ECM of holes are enhanced by optimizing the duty ratio, lateral gap and inlet pressure.

## 1. Introduction

With the rapid development of accuracy and miniaturization in the aerospace, automotive and medical industries, small holes of special shape and groove structures have appeared on many mechanical parts. For example, in order to meet the characteristics of lightweight and high temperature resistance of turbine blades, high temperature resistant cemented carbide [1,2,3] is used as the main material of blades and the film cooling holes [4,5] are machined to improve the properties of thermal dissipation. However, traditional machining is difficult to machine microstructures in high temperature resistant cemented carbides. Electrical discharge machining [6,7] and laser machining [8,9] will form the hot recast layer and micro-cracks on the metal surface. These methods will affect the accuracy and stability of holes machining.

The ECM is based on the principle of electrochemical dissolution of the anode in the electrolyte [10,11,12,13]. The surface quality of the machined small holes is good, and there is no stress concentration and surface hardening layer. The diameter of film cooling holes is usually between 1–3 mm. The tiny structure prevents the placement of sensors in the machining area to collect temperature, flow velocity and current density distribution. The heater leased by the electrochemical reaction and electric current raises the temperature of the machining area [14,15]. The flowing electrolyte acts as a conductive medium to provide an ECM environment. The renewal of electrolysis products and heat is related to the flow field distribution of the electrolyte [16]. In order to improve the accuracy and stability of ECM of small holes, experts and scholars at home and abroad have studied the distribution characteristics of the electric, flow and temperature field in the machining area under the influence of the power supply. Chai et al. [17] studied the distribution of the volume fraction of hydrogen and the temperature in the machining area of the cooling hole with an external direct current power supply. Based on the finite element electric field model of the direct current power supply, Li et al. [18] obtained the mathematical relationship between the hole forming law and the machining time under different mask diameters. Wang et al. [19] improved the flow field and machining positioning by synchronizing the pulse power supply and low frequency vibration, which reduced the stray corrosion on the sidewall of the diamond hole. T. Sathish [20] used the parameter optimization to study the effect of the duty ratio on the roundness, taper and surface roughness of the titanium alloy holes through experiments. Manpreet [21] obtained the optimal direct current voltage suitable for ECM of silicon wafers by taking overcut and taper as the output quality characteristics. Ma et al. [22] used the pulse electrochemical method to optimize the surface roughness of non-circular holes after EDM, which was decreased from 4.227 μm to 0.229 μm.

The research structure of this article is as follows: Firstly, the mathematical model of multi-physics coupling simulation of ECM small holes is described in detail in Section 2. The machining properties of the direct current and pulse ECM are compared, and the distribution of the temperature, flow and electric field with pulse ECM are studied in Section 3 according to the established geometric model of the film cooling hole. In Section 4, the changes of the temperature, current density and side removal rate under the influence of the period, duty ratio, lateral gap and pressure are introduced in detail. Finally, the experiment of ECM of small holes in nickel-based alloys was performed to verify the simulation results and optimize the duty ratio and inlet pressure. The optimized parameters can improve the machining accuracy and stability of film cooling holes.

## 2. Multi-Physics Coupling Mathematical Model and Theoretical Analysis

**Figure 1:**
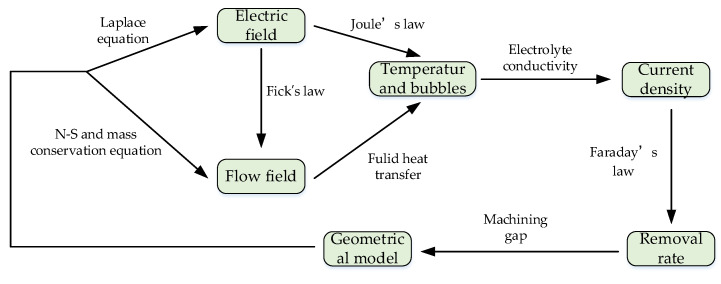
Schematic diagram of multi-physics coupling relationship.

ECM is a complex process using electrochemical principle, which involves the interaction between the electric field, flow field, heat conduction, chemical reaction and structure. Figure 1 intuitively and clearly describes the multi-physics coupling relationship in the ECM process. In this article, the pulse power supply is used for ECM of small holes. Conductivity is used as a “bridge” to connect the electric field and the flow field, so as to achieve the characteristics of the three-field coupling of heat, electricity and flow. The temperature, current density and side removal rate in the machining area under the influence of various physical fields are analyzed. In order to simplify the above model, this article makes the following assumptions:

(1) Polarization is not considered during the ECM process, and the surface of the anode material is always assumed to be in an activated state.
(2) The bubbles generated by the cathode and anode are ignored.
(3) The conductivity of the electrolyte depends only on the change of the temperature.
(4) The relative position between the electrode and the workpiece remains unchanged.

**Figure 2:**
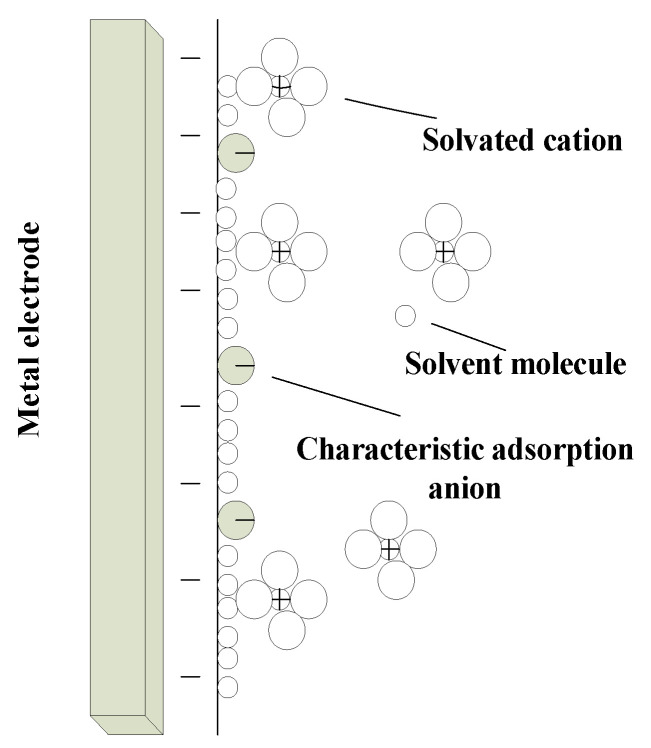
Schematic diagram of electrode system.

The electrode is not only the metal connecting the positive and negative electrodes of the power supply, but also the electrode system formed by the interface of the positive and negative electrodes and their adjacent electrolyte in the tubular electrode ECM. The details are shown in Figure 2.

When the workpiece is electrolyzed, the workpiece is made of high-temperature resistant nickel-based alloy containing carbon, silicon and other non-metallic substances. Thus, many metal and nonmetal ions are oxidized at the anode to form ions. The specific chemical equation is as follows:

M→M^n+^ + ne (1)

These ions enter the electrolyte, which are subjected to strong electric field and strong flow field inside the machining gap. They are continuously diffused towards the end face of the tubular electrode and oxidized on the cathode surface. After adsorption and deposition, it forms a nonmetallic nonconductive film layer to increase the frequency of short circuit.

### 2.1. Electric Field Model

When the ECM is in a stable state, the machining gap is maintained in a stable range. The equation of lateral gap:

(2) $$\Delta h=\frac{\eta \omega \sigma \left(U-\delta E\right)}{V_{i}}=\frac{\sigma \left(U-\delta E\right)}{i}$$

(3) $$V_{i}=\eta \omega i$$

Assuming the homogeneity of electrolyte, the potential distribution φ(*x*,*y*) in the machining gap can be expressed by the Laplace equation. The current density at any point in the electric field satisfies Ohm’s law.

(4) $$\nabla^{2}\varphi =\frac{\partial^{2}\varphi }{\partial x^{2}}+\frac{\partial^{2}\varphi }{\partial y^{2}}=0$$

(5) i=−σ·∇φ

where *U*(V) is the processing voltage, δE(V) is the voltage drop between the cathode and anode, $V_{i}$(m/s) is the material removal rate, σ(S/m) is the conductivity of the electrolyte, *η* is the current efficiency, ω(mm^3^/(A·min)) is the volume electrochemical equivalent, φ(*V*) is the electric potential and i(A/m^2^) is the local current density.

### 2.2. Flow Field Model

Assuming that the size of the bubble is small, the effect of the bubble on the electrolyte flow can be ignored. For incompressible viscous fluids, the fluid flow in the turbulent state is restricted by the Navier-Stokes equation.

(6) $$\rho \left(\frac{\partial u}{\partial t}+u\cdot \nabla u\right)=-\nabla p+\mu \Delta u$$

(7) ρ∇·u=0

where ρ(kg/m^3^) is the electrolyte density, u(m/s) is the flow velocity, p(Pa) is the pressure, μg/(m·s)) is the dynamic viscosity of the electrolyte.

Based on the change of the flow field with time, the “*k*-*ε*” turbulence model in the Reynolds average (RANS) equation is used to solve the turbulent energy “*k*” and the turbulent dissipation rate “*ε*” in the electrolyte flow process.

(8) $$\rho \frac{\partial k}{\partial t}+\rho \cdot u\cdot \nabla k=\nabla \cdot \left[\left(\mu +\frac{\mu T}{\sigma_{k}}\right)\nabla k\right]+P_{k}-\rho \varepsilon$$

(9) $$\rho \frac{\partial \varepsilon }{\partial t}+\rho \cdot u\cdot \nabla \varepsilon =\nabla \cdot \left[\left(\mu +\frac{\mu T}{\sigma_{\varepsilon }}\right)\nabla \varepsilon \right]+C_{\varepsilon 1}\frac{\varepsilon }{k}P_{k}-C_{\varepsilon 2}\rho \frac{\varepsilon^{2}}{k}$$

where $P_{k}$ is the generating term of turbulent energy, $\sigma_{k}$ and $\sigma_{\varepsilon }$ are Prandtl numbers corresponding to “*k*” and “*ε*” with values of 1.0 and 1.3, $C_{\varepsilon 1}$ and $C_{\varepsilon 2}$ are model constants with values of 1.44 and 1.92.

### 2.3. Temperature Field Model

The temperature distribution of the electrolyte is calculated by solving the internal energy, which satisfies the convection-diffusion equation.

(10) $$\rho C_{P}\frac{\partial T}{\partial t}+\rho C_{P}\mu \cdot \nabla T=\nabla \cdot \left(k_{0}\cdot \nabla T\right)+Q_{i}$$

(11) $$Q_{i}=i\cdot \nabla \varphi$$

where $C_{p}$(J/(kg · K) is the heat capacity of electrolyte and $k_{0}$ (W/(m·K) is the heat conductivity coefficient. $Q_{i}$(W/m^3^) is the Joule heat generated by the current.

The flow field and electric field affect the temperature field distribution, and the conductivity of the electrolyte is affected by the temperature. The relationship is:

(12) $$\sigma_{T}=\sigma \left(1+\gamma \left(T-T_{0}\right)\right)$$

where γ is the temperature correlation coefficient, $T_{0}$(K) is the initial temperature.

## 3. Simulation Analysis of the Temperature, Flow and Electric Field

### 3.1. The Establishment of Geometric Model

**Figure 3:**
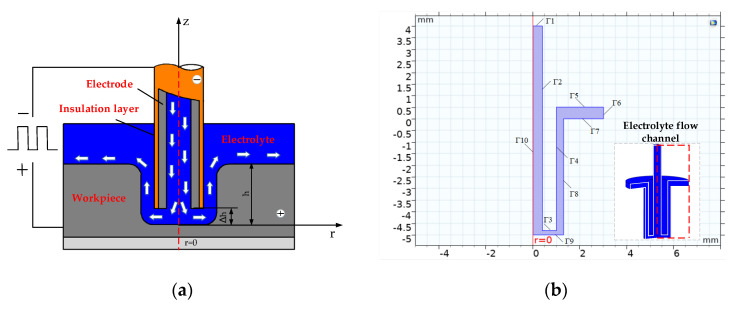
Geometric model of the film cooling hole machining; (**a**) schematic diagram of the machining zone; (**b**) electrolyte flow channel model and boundary definitions.

Figure 3 shows the geometric model of the multi-physics coupling simulation of the film cooling hole. The solution area is composed of the geometry of the electrode, contour of the workpiece and flow channel of the electrolyte. The boundaries Γ2 and Γ3 are the inner wall and front-end surface of the cathode. The boundary Γ4 is the outer wall of the cathode coated with an insulating layer. The boundaries Γ7, Γ8 and Γ9 are anodes. The boundaries Γ1 and Γ6 are the inlet and outlet of the electrolyte. The boundary Γ5 is the wall.

### 3.2. The Effect of Power Supply on the Temperature and Current Density

When the electrode and workpiece are connected to an external power supply, Joule heat is generated in the machining area. Excessive local temperature will cause electrolyte evaporation. The increase of the temperature affects the conductivity of the electrolyte and causes uneven dissolution of the workpiece surface. The pulse power supply (the pulse on is 24 V, the pulse off is 0.001 V) has a period of 1000 μs and a duty ratio of 0.5. *U(t)* is the pulse voltage. *U* is the pulse voltage amplitude. *T* is the pulse period.

(13) $$U\left(t\right)=\left\{\begin{array}{cc} flc1hs\left(\sin\left(2*pi*\frac{t}{T}\right),\frac{T}{2}\right)*U & \;t\in \left(0,\;\frac{nT}{2}\right)\\ 0.001 & t\in \left(\frac{nT}{2},\;\mathrm{nT}\right) \end{array}\right.$$

**Figure 4:**
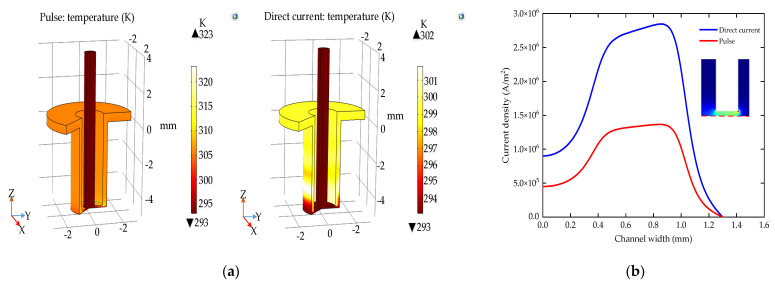
The temperature and current density with different power supply; (**a**) the temperature of the machining area; (**b**) the current density on the anode surface.

The detailed boundary conditions of the simulation model are shown in Table 1. Figure 4a shows the temperature distribution of the machining area under the direct current and pulse ECM. The abrupt change of temperature occurs at the junction of the inner-outer walls and the end face of the electrode. In the steady state, the highest temperature of the direct current machining area is 323 K, while the pulse machining area is 303 K. Figure 4b shows that the current density distribution on the surface of the workpiece is similar to the temperature distribution. In the machining area with a radius of 1 mm, the current density range is reduced from 1.95 × 10^6^ A/m^2^ with the direct current to 0.91 × 10^6^ A/m^2^ with the pulse. Therefore, the pulse ECM improves the temperature distribution by interval discharge and reduces the current density difference on the workpiece surface.

To sum up: Compared with the direct current ECM, the pulse ECM has better performance in the ECM of small holes. On the one hand, the pulse ECM avoids the effect of high temperature on the machining accuracy; on the other hand, it is beneficial to reduce the short circuit caused by uneven dissolution.

## 4. Physical Field Distribution with Pulsed Power Supply

### 4.1. Temperature Field

In order to study the effect of pulse ECM on the temperature, four reference points are selected on the electrode end face at equal intervals (the area with large temperature changes). The process parameter is a voltage of 24 V (the period is 1000 μs, the duty ratio is 0.5, the lateral gap is 0.12 mm, the pressure is 0.2 MPa and the depth is 5 mm).

**Figure 5:**
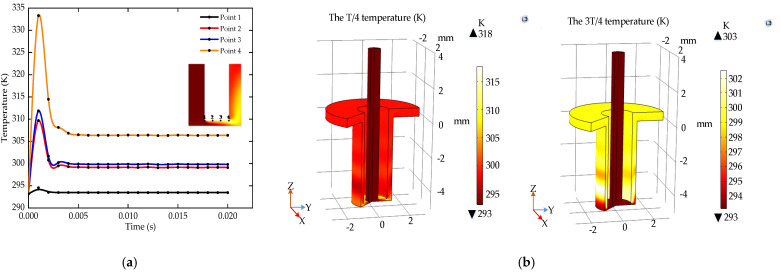
The temperature distribution of the pulse ECM; (**a**) the temperature change rules of reference points; (**b**) the temperature during a pulse period.

Figure 5a shows the temperature change rules of reference points over time. The temperature reaches the maximum in the first period. After about 5 periods, it drops to a stable state. Figure 5b shows that the temperature in the pulse ECM machining area increases to 318 K within 1/4 period, and the temperature decreases to 303 K within 3/4 period. During the pulse off, the discharge between the electrodes and the electrochemical reaction are suspended which causes no current to flow in the circuit. The cumulative effect of Joule heat generated by the current and the heat of electrochemical reaction is reduced. Therefore, the pulse ECM can reduce the influence of temperature on electrical conductivity and current density, which is beneficial to improve the stability of ECM.

### 4.2. Flow Field

In order to study the influence of the pulse ECM on the flow velocity of electrolyte, four reference points are selected equidistantly on the workpiece surface directly opposite the electrode end surface, where the flow velocity varies greatly. The process parameter is a voltage of 24 V (the period is 1000 μs, the duty ratio is 0.5, the lateral gap is 0.12 mm, the pressure is 0.2 MPa and the depth is 5 mm).

**Figure 6:**
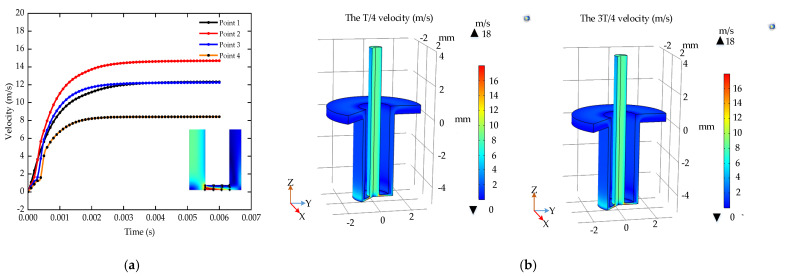
The velocity distribution of the pulse ECM; (**a**) the velocity change rules of reference points; (**b**) the velocity during a pulse period.

The velocity of reference points increases rapidly in the first period and gradually increases to a stable state after about four periods, as shown in Figure 6a,b shows that the electrolyte flow velocity varies less in a period of the pulse ECM. However, during the pulse off, the discharge between the electrodes is stopped to allow sufficient time for the electrolyte to take away the Joule heat generated by the current and the electrolysis product of electrochemical reaction. Therefore, the pulse ECM can speed up the renewal of the electrolyte and improve the machining accuracy.

### 4.3. Electric Field

According to the simulation analysis of the temperature and flow field with the pulse ECM, the stable state is about 5 periods later. The process parameter is a voltage of 24 V (the period is 1000 μs, the duty ratio is 0.5, the lateral gap is 0.12 mm, the pressure is 0.2 MPa and the depth is 5 mm).

**Figure 7:**
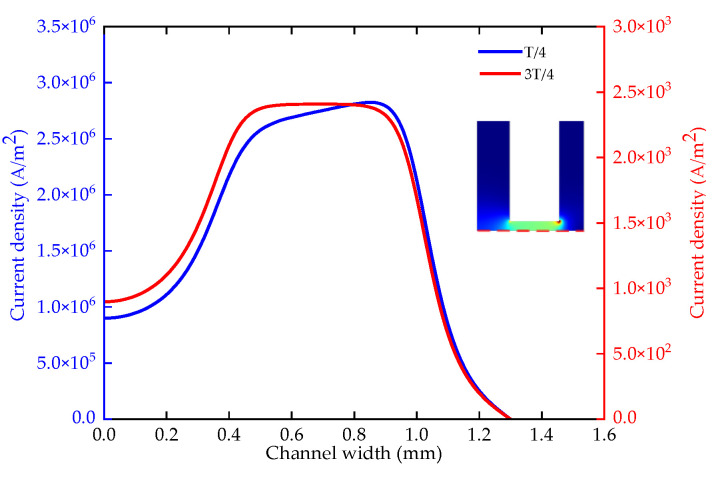
The current density of the pulse ECM.

Figure 7 shows the current density on the workpiece surface within 1/4 and 3/4 period during a stable machining period. In the machining area with a radius of 1 mm, the current density range at the T/4 is 1.7 × 10^6^ A/m^2^. The current density range of the 3T/4 is 1500 A/m^2^. The range of current density in a period is 0.91 × 10^6^ A/m^2^. The current density on the workpiece surface is larger in the center and smaller on both sides. It is easy to form a “bulge” structure at the bottom during the ECM. The pulse ECM reduces the current density range, which is beneficial to the uniform dissolution of the workpiece. Therefore, the pulse ECM can reduce the short circuit caused by the “bulge” structure and improve the stability of the machining.

## 5. Influence of Process Parameters

### 5.1. The Effect of Pulse Period

**Figure 8:**
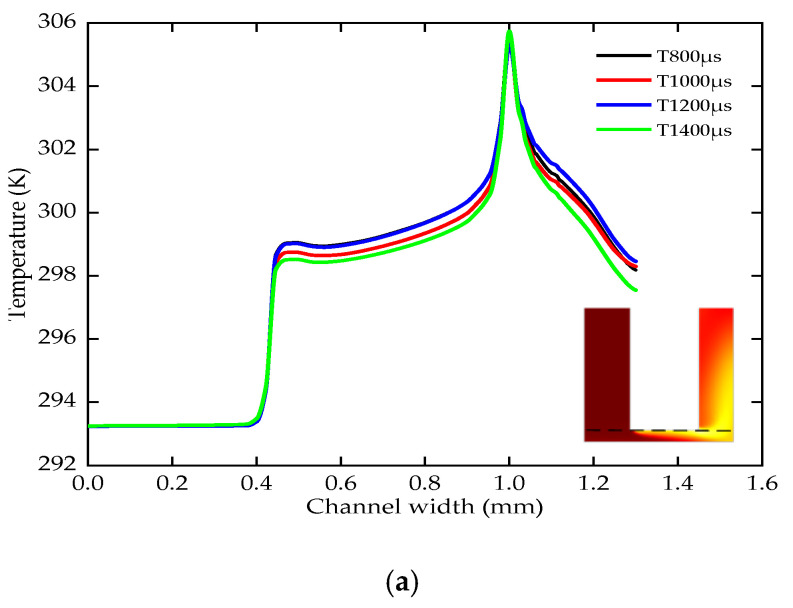

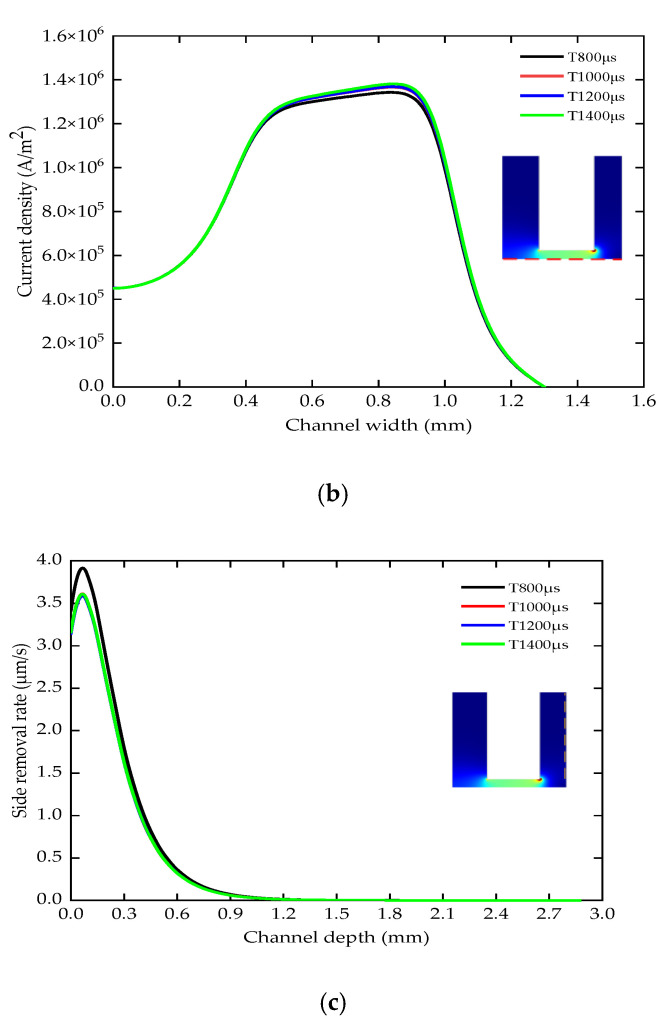
The temperature, current density and side removal rate of various pulse periods; (**a**) the change of the temperature; (**b**) the change of the current density; (**c**) the change of the side removal rate.

The pulse period is 800–1400 μs (the voltage is 24 V, the duty ratio is 0.5, the lateral gap is 0.12 mm, the pressure is 0.2 MPa, the depth is 5 mm). In the steady state, the temperature distribution of the electrode end surface is basically the same with different pulse periods, as shown in Figure 8a. The temperature has two steps at 0.4 mm and 1 mm. The highest temperature is 306 K. With different pulse periods, the average current density of a period on the workpiece surface is basically the same, as shown in Figure 8b. The maximum current density is about 1.36 × 10^6^ A/m^2^.

Figure 8c shows the side removal rate of the work piece. The maximum side removal rate for a period of 800 μs is approximately 3.9 μm/s. The side removal rate in the other cycles are basically the same, and the maximum side removal rate is 3.5 μm/s. At the same duty ratio, the proportion of discharge time within the whole pulse ECM process remains unchanged. While more heat and electrolytic products are generated during the pulse on, there is also enough time to update the Joule heat and product within the pulse off. Therefore, the temperature, current density and side removal rate fluctuate in a small range. The period change has little effect on machining accuracy and stability in the pulse ECM.

### 5.2. The Effect of Duty Ratio

**Figure 9:**
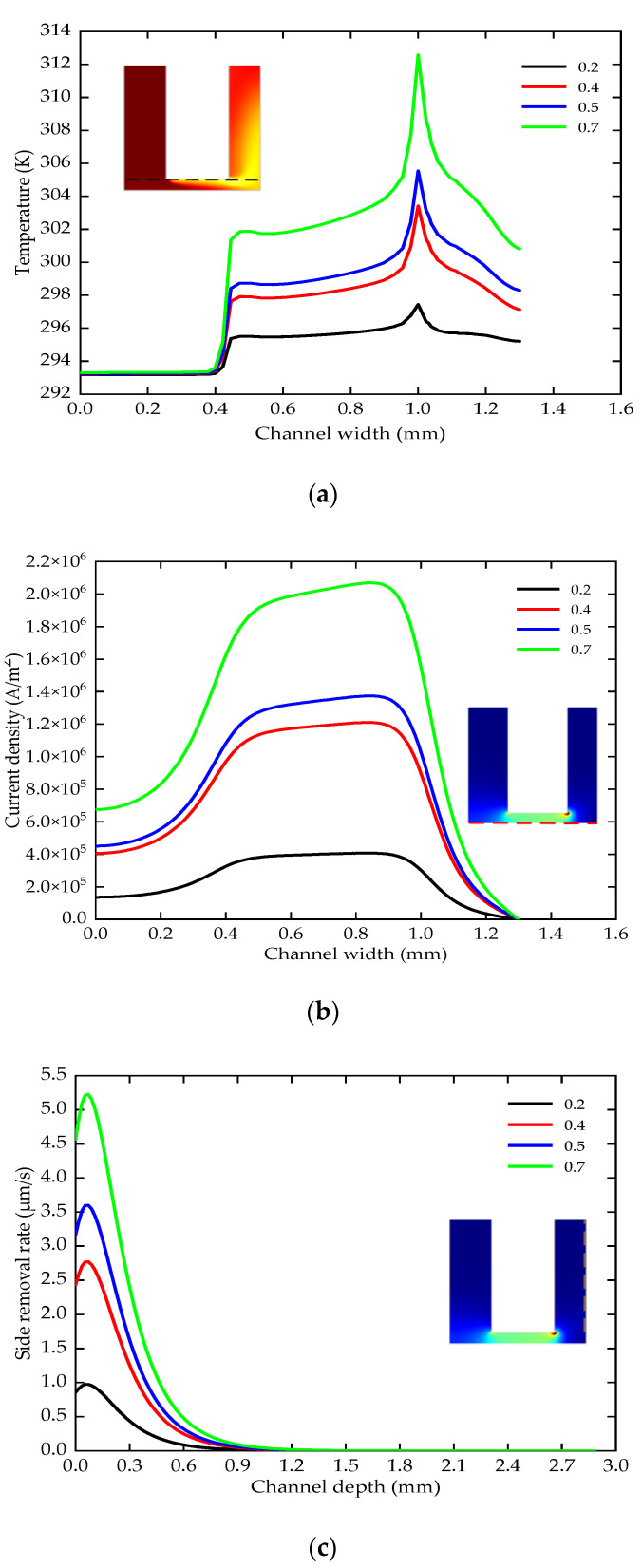
The temperature, current density and side removal rate of various duty ratios; (**a**) the change of the temperature; (**b**) the change of the current density; (**c**) the change of the side removal rate.

The duty ratio is 0.2–0.7 (the voltage is 24 V, the period is 1000 μs, the lateral gap is 0.12 mm, the pressure is 0.2 MPa, the depth is 5 mm). In the steady state, the temperature distribution of the electrode end surface is shown in Figure 9a. As the duty ratio increases, the two temperature steps also become larger. The maximum temperature increased from 297 K to 313 K. The rise of the temperature will increase the conductivity of the electrolyte, which in turn affects the distribution of the current density in the entire machining area. Figure 9b shows the current density on the workpiece surface. Due to the uneven distribution of temperature and different duty ratios, the maximum current density increases from 0.4 × 10^5^ A/m^2^ to 2.06 × 10^6^ A/m^2^ in the machining area with a radius of 1 mm. Excessive current density range will cause uneven dissolution of the surface of the work piece.

It can be seen from Figure 9c that the side removal rate and dissolution area change greatly. When the duty ratio is 0.7, the maximum side removal rate is 5.1 μm/s and the dissolution area is 1.5 mm. However, when the duty ratio is 0.2, the maximum side removal rate is 1 μm/s, and the dissolution area is 0.9 mm. If the duty ratio is too small, the machining efficiency will decrease. The duty ratio is too large, and the increase of temperature and side wall dissolution area will affect the machining accuracy. Therefore, the medium duty ratio is conducive to improving the machining accuracy and stability.

### 5.3. The Effect of Lateral Gap

**Figure 10:**
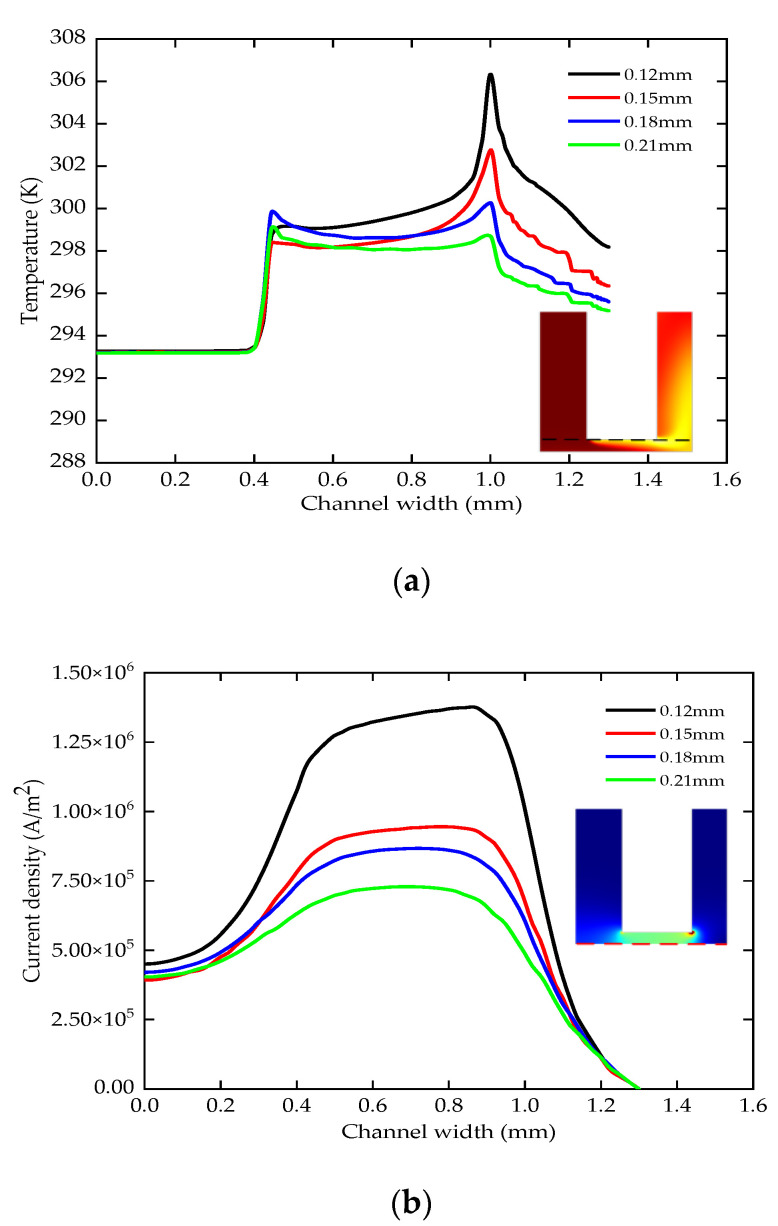

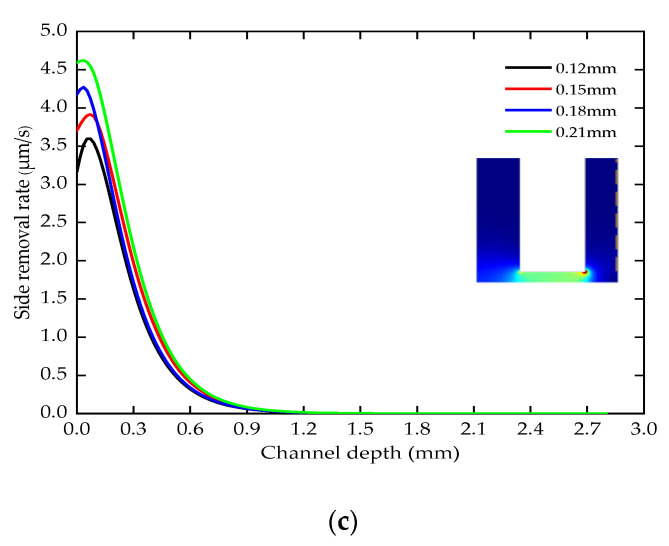
The temperature, current density and side removal rate of various lateral gaps; (**a**) the change of the temperature; (**b**) the change of the current density; (**c**) the change of the side removal rate.

The lateral gap is 0.12–0.21 mm (the voltage is 24 V, the period is 1000 μs, the duty ratio is 0.5, the pressure is 0.2 MPa, the depth is 5 mm). The increase of the lateral gap reduces the temperature step at a radius of 1 mm. The maximum temperature of the electrode end surface decreases from 307 K to 299 K in the steady state, as shown in Figure 10a. As the lateral gap increases, Figure 10b shows that the current density range decreases from 0.92 × 10^6^ A/m^2^ to 0.43 × 10^6^ A/m^2^ in the machining area with a radius of 1 mm.

It can be seen from Figure 10c that the side removal rate increases uniformly according to the increase of the lateral gap, and the dissolution area does not change significantly. Therefore, the larger lateral gap accelerates the electrolyte and product updates per unit time. It is conducive to improving the temperature and current density distribution in the machining area and can ensure the stability of machining.

### 5.4. The Effect of Inlet Pressure

**Figure 11:**
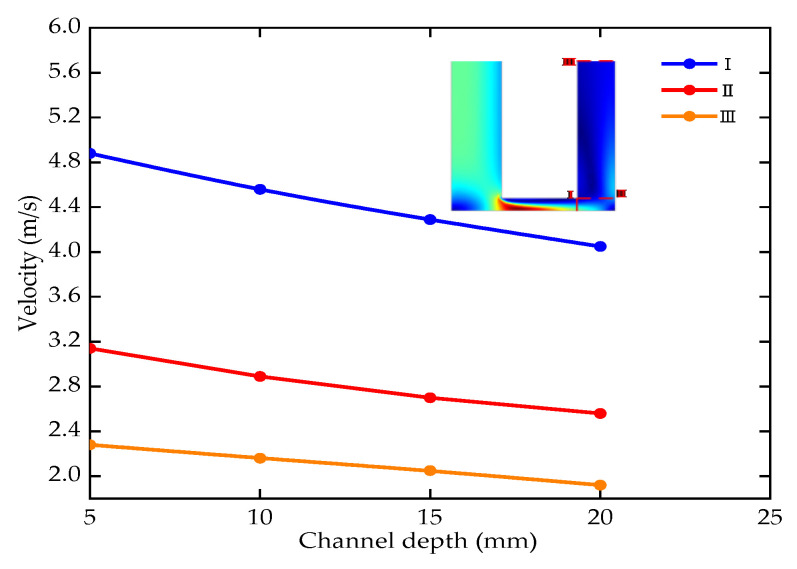
Electrolyte flow velocity with different machining depths.

The machining depth is 5–20 mm (the voltage is 24 V, the period is 1000 μs, the duty ratio is 0.5, the pressure is 0.2 MPa, the lateral gap is 0.18 mm). Three cut-off lines were selected along the flow direction of the electrolyte to obtain the distribution of the average flow velocity of the electrolyte at various machining depths, as shown in Figure 11.

The average velocity decreases with the increase of electrolyte flow distance and the machining depth. The electrochemical dissolution of materials generates a large amount of heat and electrolysis products in the machining area. The decrease of flow velocity will reduce the heat and product update rate. In serious cases, the direct contact between the electrode and the workpiece results in a short circuit which affects the stability of the machining. Therefore, it is essential to improve the flow field distribution of machining gap.

**Figure 12:**
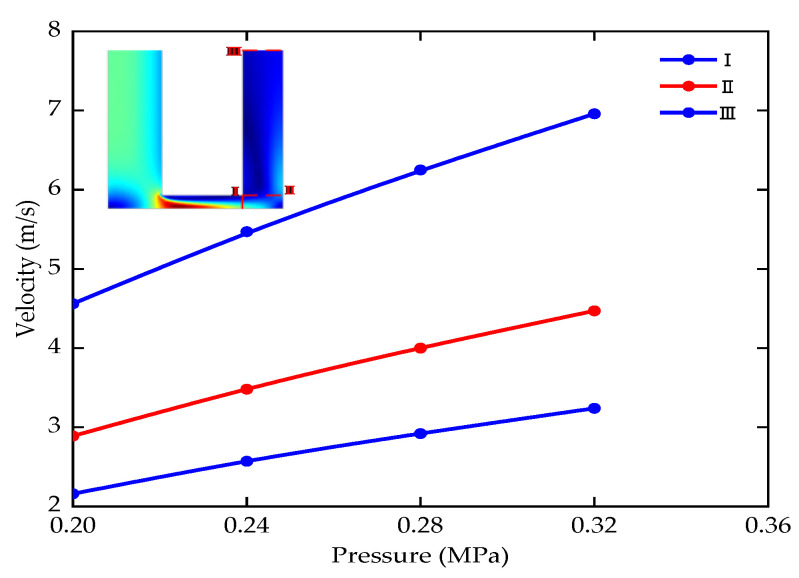
Electrolyte flow velocity with the different inlet pressure.

The inlet pressure is 0.2–0.32 MPa (the voltage is 24 V, the period is 1000 μs, the duty ratio is 0.5, the machining depth is 5–20 mm, the lateral gap is 0.18 mm). The flow velocity changes in the machining area are shown in Figure 12.

The average flow velocity of the electrolyte at the cut-off line increases steadily when the inlet pressure is appropriately increased according to different machining depths. If the inlet pressure is too small, the electrolyte flow velocity is too low to take away the electrolytic products in time. The inlet pressure is too large, and the rapid increase of flow velocity is easy to cause vortex. Therefore, appropriately increasing the inlet pressure can improve the flow field distribution and the stability of the ECM of small holes.

## 6. Experimental Results and Discussion

### 6.1. Experimental Equipment

**Figure 13:**
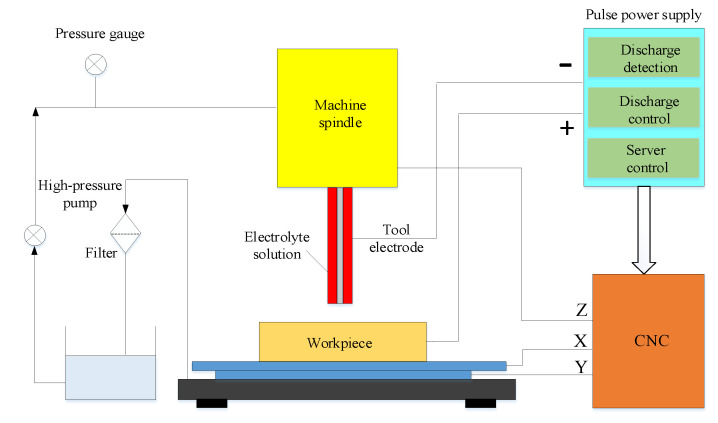
Schematic diagram of ECM system.

The multi-physics coupling simulation results and the optimization of machining parameters are verified by experiments of the ECM holes. The schematic of the machining system is shown in Figure 13, including electrolyte update system, pulse power supply and CNC system. The workpiece is driven by a stepping motor to move along the X–Y plane. The tool electrode connected to the spindle feeds uniformly along the motor-driven linear guide in the Z direction. The electrolyte is pumped to the machining area through the pressure in the flow channel. The negative and positive electrodes of the pulse power supply are, respectively, connected to the tool electrode and the workpiece. All experiments were performed using this systemin this study.

### 6.2. Experimental Materials

The tool electrode is a titanium tube coated with PTFE, and the thickness of the insulating layer is about 40 μm. The tool electrode has the inside diameter of 1.8 mm and outside diameter of 2.1 mm. The electrolyte is a 13% nitric acid solution. The voltage of the pulse power supply is 24 V. The workpiece material is a high-temperature resistant GH4169 nickel-based alloy, as shown in Table 2.

**Figure 14:**
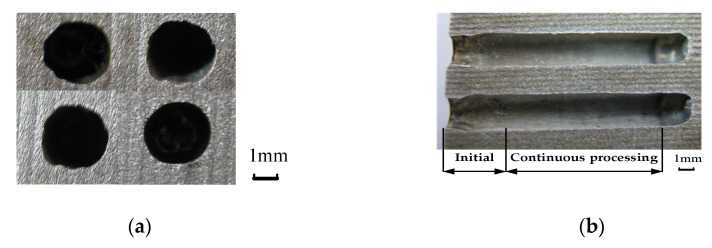
The morphology of the machined holes; (**a**) profile of small holes inlet end; (**b**) profile of keyhole section.

The sampling morphology of the entrance of the ECM small holes is shown in Figure 14a. The axial section of the machined workpiece is shown in Figure 14b, which describes the change of aperture morphology.

By analyzing Figure 14, it can be found that the entrance end is approximately circular. Since the electrolyte is sprayed to the workpiece surface by the tubular electrode in the initial machining stage and the scattering area is wide, the gap electric field is stray. It results in the difference between the section morphology of the inlet end and that of the stable machining section.

In order to study the relationship between the morphology of the inlet end and the machining gap and the electric field, a comparative experiment was carried out, as shown in Table 3. A tool electrode feed rate of 0 m/s is a special case. The initial machining gap is set. The tool electrode remains stationary throughout the ECM process from the beginning to the end of the reaction.

### 6.3. Analysis of Processing Quality

Experiments compare the dimensional accuracy and machining efficiency of the ECM holes at various duty ratios and inlet pressure. The single side gap and linear removal rate are used as evaluation indicators. For the accuracy of the comparison test, other variables are defined as constants.

The single side gap is the difference between the radius of the machining hole and the radius of the tool electrode.

(14) $$D=\frac{1}{n}\sum_{i=1}^{n}\left(r_{h}-r_{0}\right)$$

where $r_{h}$(mm) is the radius of the machining hole,$\;r_{0}$(mm) is the radius of the tool electrode, *n* is the number of measurements of the diameter of the hole along the depth direction.

The linear removal rate is the depth of the electrochemically machined hole per unit time.

(15) $$L=\frac{m}{\rho *t*\bar{A}}$$

where *m*(kg) is the mass of removal, ρ(kg/m^3^) is the density of the workpiece, *t*(s) is the machining time and $\bar{A}$(m^2^) is the average value of the cross-section of the small hole.

**Figure 15:**
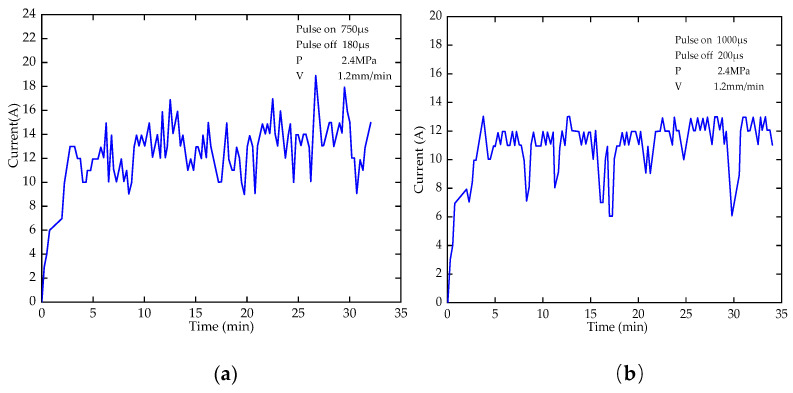
Current acquisition diagram; (**a**) sampling current with pulse on 750 μs and pulse off 180 μs; (**b**) sampling current with pulse on 1000 μs and pulse off 200 μs.

The specific machining current collected in the test in Table 3 is shown in Figure 15a,b. The change law of acquisition current with time is the same with different process parameters.

Analysis shows that the inlet current fluctuates. The current tends to be constant as the machining proceeds. According to Sander’s equation, the details are shown as follows:

(16) $$\frac{i\tau^{\frac{1}{2}}}{{C_{o}}^{*}}=\frac{nFAD_{O}^{\frac{1}{2}}\pi^{\frac{1}{2}}}{2}$$

where:

*I*—Current (A)
*τ*—Transition time of potential (S)
*F*—Faraday constant (C)
*A*—Electrolysis Area (cm^2^)
*D*o—Diffusion coefficient of electrolytic reaction material (cm^2^/s)
C_o_*—Surface concentration of the reactive substance at the electrode (mol/L).

When the machining enters a stable state, the processing current *I* is inversely proportional to *τ* and directly proportional to C_o_*, *D_o_* and *A*. The parameters are relatively stable and the machining current tends to be constant. According to the analysis of Figure 15, the stationary stage of machining conforms to the description of current in the De Sang equation. In the machining inlet stage, the instability of C_o_*, *D_o_* and *A* will result in the unstable current and the uneven morphology of the inlet section.

### 6.4. Experimental Results and Discussion

**Figure 16:**
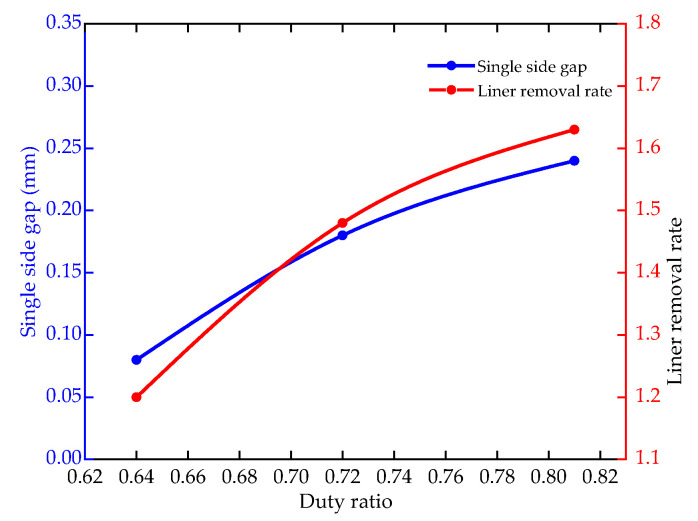
The single side gap and linear removal rate at various duty ratios.

Figure 16 shows the single side gap and linear removal rate of the hole with duty ratios of 0.64–0.82. Process parameters: The inlet pressure is 2.7 MPa, the pulse period is 1000 μs, the depth is 40 mm, the lateral gap is 0.2 mm. As the duty ratio increases, the single side gap increases from 0.08 mm to 0.24 mm, and the linear removal rate increases from 1.2 mm/min to 1.63 mm/min. In the simulation analysis, the current density of the workpiece surface and the side removal rate also increase with the increase of the duty ratio. Therefore, the simulation results and experiments are mutually verified, and choosing a medium duty ratio is beneficial to improve the machining accuracy.

**Figure 17:**
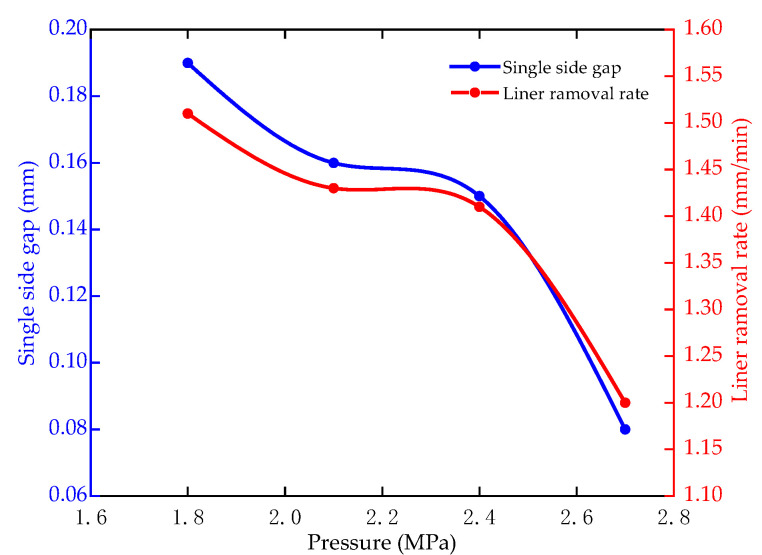
Single side gap and linear removal rates with different duty ratios.

Figure 17 shows the single side gap and linear removal rate of the hole with the inlet pressure of 1.8–2.7 MPa. Process parameters: the pulse period is 1000 μs, the duty ratio is 0.64, the depth is 40 mm and the lateral gap is 0.2 mm. The initial inlet pressure is 1.8 Mpa. For every 10 mm increase in machining depth, the inlet pressure increases by 0.3 MPa. The single side gap is reduced from 0.19 mm to 0.08 mm. The linear removal rate is reduced from 1.51 mm/min to 1.2 mm/min. In the simulation analysis, the electrolyte flow velocity increases with the increase of pressure. The faster flow velocity accelerates the removal of Joule heat and products, which reduces the uneven dissolution of the sidewall caused by the high temperature. Therefore, increasing the inlet pressure is beneficial to improve the forming accuracy of holes for larger machining depths.

## 7. Conclusions

In this article, a study of multi-physics coupling simulation of film cooling holes is established. The stability and machining accuracy of the direct current and pulse ECM are compared. Through the research and experimental verification of the electrical parameters (period, duty ratio) and process parameters (lateral gap, pressure) of the pulse ECM, the following conclusions are drawn:

(1) The current density is more evenly distributed and the temperature stage in the machining area is reduced with the pulse ECM.
(2) The temperature, current density and side removal rate gradually increase with the increase of the duty cycle but are not affected by the period. The use of medium duty ratio helps to improve the machining accuracy of holes.
(3) The larger lateral gap can reduce the range of current density and accelerate the renewal of Joule heat and electrolytic products.
(4) Increasing the inlet pressure is beneficial to improve the flow field distribution of machining gap and reduce the single side gap, which improve the accuracy and stability of ECM holes.
(5) The reasons for the differences in the morphology of the entrance section of the machined small holes were analyzed. The electrolyte injection range and initial machining gap of the initial stage affect the electric field distribution in the machining area.

## Figures and Tables

**Table 1:** Parameters of the simulation model.

Parameters	Values
Density of electrolyte (*ρ*)	1200 (kg/m^3^)
Dynamic viscosity of electrolyte (μ)	0.001 (Pa/s)
Heat capacity of electrolyte ( $C_{p}$ )	4200 (J/(kg·K)
Initial conductivity (σ)	12 (S/m)
Heat conductivity coefficient ( $k_{0}$ )	0.65 (W/(m·K))
Pulse period (T)	800, 1000, 1200, 1400 (μs)
Duty ratio (1)	0.2, 0.4, 0.5, 0.7
Lateral gap (∆h)	0.12, 0.15, 0.18, 0.21 (mm)
Inlet pressure ( $p_{0}$ )	0.2 (MPa)
Temperature correlation coefficient (γ)	0.16
Volume electrochemical equivalent (ω)	2 ${\mathrm{mm}}^{3}$ /(A·min))

**Table 2:** Composition table of high temperature nickel-based alloy GH4169.

Composition	N_i_	C_r_	Mo	Cu	Ti	Al	Nb	C
Percentage	55	21	3.3	0.3	1.15	0.7	5.5	0.06

**Table 3:** Validation experiments.

No	Ton (μs)	Toff (μs)	V (mm/min)	gs (mm)
1	1850	500	0	3.0
2	1850	500	1.02	2.5
3	1850	500	1.14	2.4
4	1850	500	1.26	2.3
5	2000	500	0	3.1
6	2000	500	1.02	2.6
7	2000	500	1.14	2.5
8	2000	500	1.26	2.4

## Data Availability

Data is contained within the article.

## Abbreviations

ECM	Electrochemical Machining
CNC	Computer Numerical Control
PTFE	Polytetrafluoroethylene
